# The Efficacy of Intra-Arterial Plus Intravesical Chemotherapy *Versus* Intravesical Chemotherapy Alone After Bladder-Sparing Surgery in High-Risk Bladder Cancer: A Systematic Review and Meta-Analysis of Comparative Study

**DOI:** 10.3389/fonc.2021.651657

**Published:** 2021-05-27

**Authors:** Zhongbao Zhou, Yuanshan Cui, Shuangfeng Huang, Zhipeng Chen, Yong Zhang

**Affiliations:** ^1^ Department of Urology, Beijing TianTan Hospital, Capital Medical University, Beijing, China; ^2^ Department of Urology, The Affiliated Yantai Yuhuangding Hospital of Qingdao University, Yantai, China; ^3^ Second Clinical Medical College, Binzhou Medical University, Yantai, China; ^4^ Department of Urology, Weifang People’s Hospital, Weifang, China

**Keywords:** intra-arterial chemotherapy, intravesical chemotherapy, bladder-sparing surgery, high-risk bladder cancer, meta-analysis

## Abstract

**Background:**

Due to the poor prognosis, the treatment of high-risk bladder cancer (HRBC) remains controversial. This meta-analysis aims to access the efficacy of intra-arterial chemotherapy (IAC) combined with intravesical chemotherapy (IC) *versus* IC alone after bladder-sparing surgery in HRBC.

**Methods:**

A systematic search of PubMed, Cochrane Library databases, EMBASE (until June 2020) was conducted. PRISMA checklist was followed. The data were analyzed by RevMan v5.3.0.

**Results:**

A total of five articles including 843 patients were studied. The analysis demonstrated that the IAC + IC group had a greater improvement of overall survival (P = 0.02) and significant reduction in terms of tumor recurrence rate (P = 0.0006) and tumor progression rate (P = 0.008) compared with the IC group. The recurrence-free survival in the IAC + IC group was significantly higher than that in the IC group (P = 0.004), but there was no significant difference in progression-free survival between the two groups (P = 0.32). In addition, the combination of IAC and IC significantly extended tumor recurrence interval (P = 0.0001) and reduced tumor-specific death rate (P = 0.01) for patients with HRBC compared with IC alone. For side effects related with IAC, although about half of the patients experienced some toxicities, most of them were mild and reversible (grades 1–2, 22.3% *vs*. grade 3–4, 2.7%), mainly including nausea/vomiting (P = 0.0001), neutropenia (P = 0.002), and alanine aminotransferase (P = 0.0001).

**Conclusion:**

Patients with HRBC treated with IAC + IC after bladder-sparing surgery had a marked improvement in the overall survival, recurrence-free survival, time interval to first recurrence, tumor recurrence rate, tumor progression rate, and tumor-specific death rate than patients treated with IC alone. However, progression-free survival was not significantly correlated with treatment strategy. In addition, patients seemed to tolerate well the toxicities related with IAC.

**Systematic Review Registration:**

PROSPERO, identifier CRD42021232679.

## Introduction

Bladder cancer (BC) is the seventh most common cancer in men with age standardized incidence rate of nine (1). Among them, non-muscle invasive bladder cancer (NIMBC) is a group of heterogeneous tumors, and its biological potential is limited to the bladder urothelium or lamina propria, which accounts for 75% of all cases of BC. A quarter of patients with NIMBC are stage T1 grade 3 (T1G3) BC, and they have the worst prognosis among NIMBC, with 5-year recurrence rate and progression rate of 41 and 20% respectively (2, 3). Due to poor prognosis, the treatment of high-risk BC (HRBC) remains controversial.

Early radical cystectomy (RC) after the first diagnosis is seen as an over-treatment, because it requires urinary diversion which has a negative impact on the quality of life. Therefore, many people are reluctant to accept RC as the first-line treatment of HRBC (4–6). Transurethral resection of bladder tumor (TURB) followed by instillation of Bacillus Calmette–Guerin (BCG) is a standard treatment recommended by the guidelines of the American Urological Association for HRBC in recent decades (7). However, because of the constraints of price and availability, the application of BCG in developing countries is limited. Therefore, to lower the risk of recurrence and progression of HRBC, new treatments are needed. Recently, the use of intra-arterial chemotherapy (IAC) *via* the bladder feeding artery after bladder-sparing therapy for HRBC has increased, and its side effects were less than adjuvant chemotherapy, which is an effective method to reduce the postoperative recurrence and progression rate (8–10). Several studies have evaluated the efficacy of IAC combined with IC *vs* IC alone after bladder-sparing surgery for patients with HRBC.

At present, there is still a lack of evidence-based medicine to explore prognostic outcomes between IAC + IC and IC alone after bladder-sparing surgery for HRBC. To the best of our knowledge, the present study is the first meta-analysis to compare their therapeutic effects.

## Materials and Methods

### Protocol

The Preferred Reporting Items for Systematic Reviews and Meta-Analyses (PRISMA) checklist was used as the guideline (11). And this study was registered at PROSPERO (CRD42021232679).

### Information Sources and Literature Search

The search was processed in the PubMed, EMBASE, and the Cochrane Controlled Trials Register (until June 2020), using various combinations of keywords including IAC, IC, and bladder cancer (bladder tumor). The study was limited to published articles with no restrictions on language. The references of related articles were also searched. Two authors independently performed the study selection (ZZ and CY). Full-text review was required where titles and abstracts were insufficient to determine if the study met the inclusion criteria. One author (HS) performed data extraction with independent verification performed by another author (CZ). Disagreements were resolved by consensus.

### Inclusion Criteria, Exclusion Criteria, and Trial Selection

**Figure 1:**
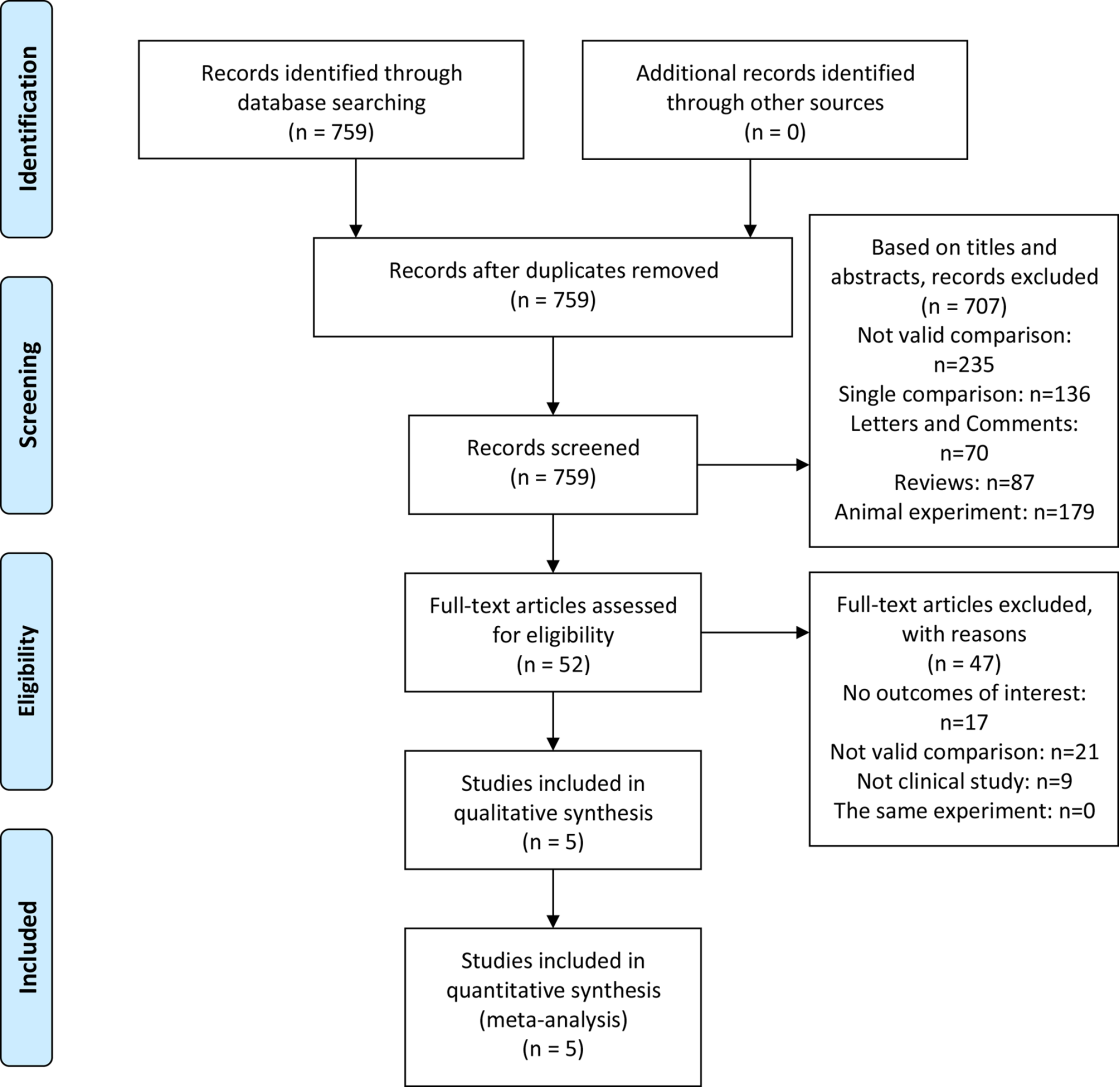
PRISMA of selection process.

The inclusion criteria were as follows: (a) Population: NMIBC patients undergoing TURBT; (b) Intervention and comparator: IAC plus IC *vs* IC alone after bladder-sparing surgery for HRBC was evaluated; (c) Outcomes: overall survival (OS), tumor recurrence rate (TRR), tumor progression rate (TPR), recurrence-free survival (RFS), progression-free survival (PFS), tumor recurrence interval (TRI), tumor-specific death rate (TSDR), and IAC related adverse events (AEs) (including nausea/vomiting, hypoleukemia, neutropenia, alanine aminotransferase, and creatinine); (d) Study designs: clinical trials. Exclusion criteria were as follows: not clinical trials, such as abstract, review, comment, or animal experiment. Criteria for included studies based on PICOS structure (**Table 1**) (12). The flow diagram of the study is presented in **Figure 1**.

**Table 1:** Criteria for included studies based on PICOS Structure.

	Population	Intervention	Comparator	Outcomes	Study Designs
Inclusion criteria	NMIBC patients (Ta/T1) undergoing TURBT.	IAC + IC	IC	Overall survival, tumor recurrence rate, tumor progression rate, recurrence-free rate, progression-free survival, tumor recurrence interval, tumor-specific death rate, and IAC related adverse events.	Clinical research.
Exclusion criteria	Stage 2 or higher tumors, previous treatment with any kind of instillation therapy, and any another concomitant malignancy.	Other therapy.	Other therapy.	Qualitative outcomes such as patient feelings; Inadequate indicators.	Letters, comments, reviews, and animal experiment.

NMIBC, non-muscle-invasive bladder cancer; TURBT, transurethral resection of bladder tumor; IAC, intra-arterial chemotherapy; IC, intravesical chemotherapy.

### Quality Assessment Methods

The Cochrane Handbook and Newcastle-Ottawa Scale (NOS) score were used to analyze the quality assessment (13, 14). Every article was evaluated by three quality classification standards: (+) low possibility of bias; ()? secondary possibility of bias; (–) high possibility of bias. The quality of retrospective study was evaluated using NOS score (14). The study defined it as three levels: score of seven or more for low risk of bias, score of four to six for moderate risk of bias, and score of lower than four for high risk of bias, respectively.

### Data Acquisition

The following data were extracted from the included studies: (a) study design; (b) first author’s name and date of study; (c) sample size, treatment and median follow-up period; (d) gender (male/female), tumor size (<3 cm/≥3 cm), number of tumors (single/multifocal), treatment protocol, and eligibility criteria; (e) OS, TRR, TPR, RFS, PFS, TRI, TSDR, and IAC related AEs (including nausea/vomiting, hypoleukemia, neutropenia, alanine aminotransferase, and creatinine). The main outcome of interest for this study was OS. Secondary outcomes were TRR, TPR, RFS, PFS, TRI, TSDR, and IAC related AEs.

The primary recurrence endpoint was defined as the reoccurrence of tumor at any grade and any stage during follow-up. The end point for a patient in the study was the time when bladder cancer recurrence and tumor progression (recurrence with stage T2 or higher) had been found and histologically confirmed. RFS was defined as the date of transurethral resection (TUR) to the date of first documented clinical recurrence. Progression was defined as the confirmation of muscle-invasive lesions (T2), invasion beyond the bladder tissue (T3/T4), or metastatic disease by tissue biopsy. PFS was defined as the date of TUR to the date of first documented clinical progression and metastasis. OS was defined as the date from diagnosis until the date of death resulting from any cause.

### Statistical Analysis

The analysis of the study was performed using Review Manager version 5.3.0 (15). The analysis used mean difference (MD) with 95% confidence intervals (CIs) to evaluate continuous data, and the odds ratio (OR) with 95% CI was used to evaluate dichotomous data. We measured the magnitude of heterogeneity with Cochrane’s Q tests and I^2^ statistics. P-value ≤0.05 or I^2^ ≥50% reflected a significant heterogeneity between the studies. To reduce the heterogeneity, a random-effects model was used in the study. All adverse reactions were scored with CTCAE v4.0. Grade referred to the severity of adverse events. CTCAE v4.0 showed grades 1 to 5. According to the following guidelines, a unique clinical description of the severity of each AE was made: Grade 1 (mild AE), grade 2 (moderate AE), grade 3 (severe AE), grade 4 (life-threatening AE), grade 5 (AE related death).

## Results

### Study Selection and Characteristics of the Trials

A total of 759 articles were initially retrieved from the databases. On the basis of the abstracts and titles, 707 studies were excluded. Due to a lack of effective data, 47 studies were excluded. Five studies (9, 10, 16–18) were used to access the effect of IAC plus IC *vs* IC alone after bladder-sparing surgery for HRBC. A flowchart was presented in **Figure 1**. The basic characteristics of the five studies are presented in **Table 2**.

**Table 2:** The details of each included study.

Study	Study design	Treatment		Sample size		Median follow-up period (months, range)	Date of study	Mean age, years (SD or range)		Gender (Male/Female)		Tumor size ( <3 cm/≥3 cm)		Number of tumors (Single/Multifocal)		Treatment protocol		Eligibility criteria
		Exp	Con	Exp	Con			Exp	Con	Exp	Con	Exp	Con	Exp	Con	IAC	IC
Lian F (18)	Retrospective cohort study	IAC + IC	IC	99	50	24.25 (5–50)	Jun 2005 to Jun 2015	60.65 ± 12.64	63.30 ± 12.79	92/7	47/3	59/40	35/15	36/63	23/27	Epirubicin (50 mg/m^2^) and cisplatin (60 mg/m^2^); Once every 4–6 weeks; Three cycle;	Epirubicin (50 mg/50 ml) immediately after TURBT; Weekly for 4–8 weeks and monthly for 6–12 months;	NMIBC patients undergoing TURBT.
Huang B (a) (16)	Prospective randomized study	IAC+IC	IC	53	98	79 (7–131)	Jan 2007 to Dec 2012	68 (30–84)	67 (29–82)	47/6	87/11	25/28	50/48	34/19	59/39	Cisplatin (60 mg/m^2^) and pirarubicin (50 mg/m^2^); Four times with 1-month interval;	Pirarubicin immediately after TURBT; Weekly for 8 weeks and monthly for 10 months;	Patients undergoing TURBT, pathologically confirmed high-grade T1 bladder transitional cell carcinoma.
Huang B (b) (17)	Retrospective cohort study	IAC+IC	IC	69	131	98 (NA)	NA	62 (30-80)	64 (29–83)	62/7	110/21	31/38	69/62	43/26	73/58	Cisplatin (60 mg/m^2^) and epirubicin (50 mg/m^2^); Four times with 1-month interval;	Pirarubicin immediately after TURBT; Weekly for 8 weeks and monthly for 10 months;	Patients undergoing TURBT, pathologically confirmed high-grade T1 G3 bladder cancer.
Sun F (10)	Prospective randomized study	IAC+IC	IC	141	142	46.9 (13–78)	Jan 2009 to Dec 2013	69.59 ± 11.02	69.0 ± 11.01	105/36	103/39	92/49	88/54	89/52	82/60	Cisplatin (50 mg/m^2^) and epirubicin (30 mg/m^2^); Three courses at 4-week intervals;	Epirubicin (50 mg) immediately after TURBT; Weekly for 8 weeks and monthly for 12 months;	Patients who were clinically diagnosed with HRBC and had no distant metastases.
Chen J (9)	Prospective randomized study	IAC+IC	IC	29	31	22 (5–58)	Jul 2006 to Dec 2011	63 (30–80)	65 (29–83)	24/5	26/5	16/13	20/11	18/11	22/9	Cisplatin (60 mg/m^2^) and epirubicin (50 mg/m^2^); Once every 4-6 weeks;	Epirubicin (50 mg/m^2^) immediately after TURBT; Weekly for 8 weeks and monthly for 8 months;	Patients had histologically confirmed T1 G3 bladder cancer.

IAC, intra-arterial chemotherapy; IC, intravesical chemotherapy; SD, standard deviation; TURBT, transurethral resection of bladder tumor; NMIBC, non-muscle-invasive bladder cancer; HRBC, high-risk non-muscle invasive bladder cancer; Exp, experimental; Con, control; NA, no available.

### Risk of Bias

**Figure 2:**
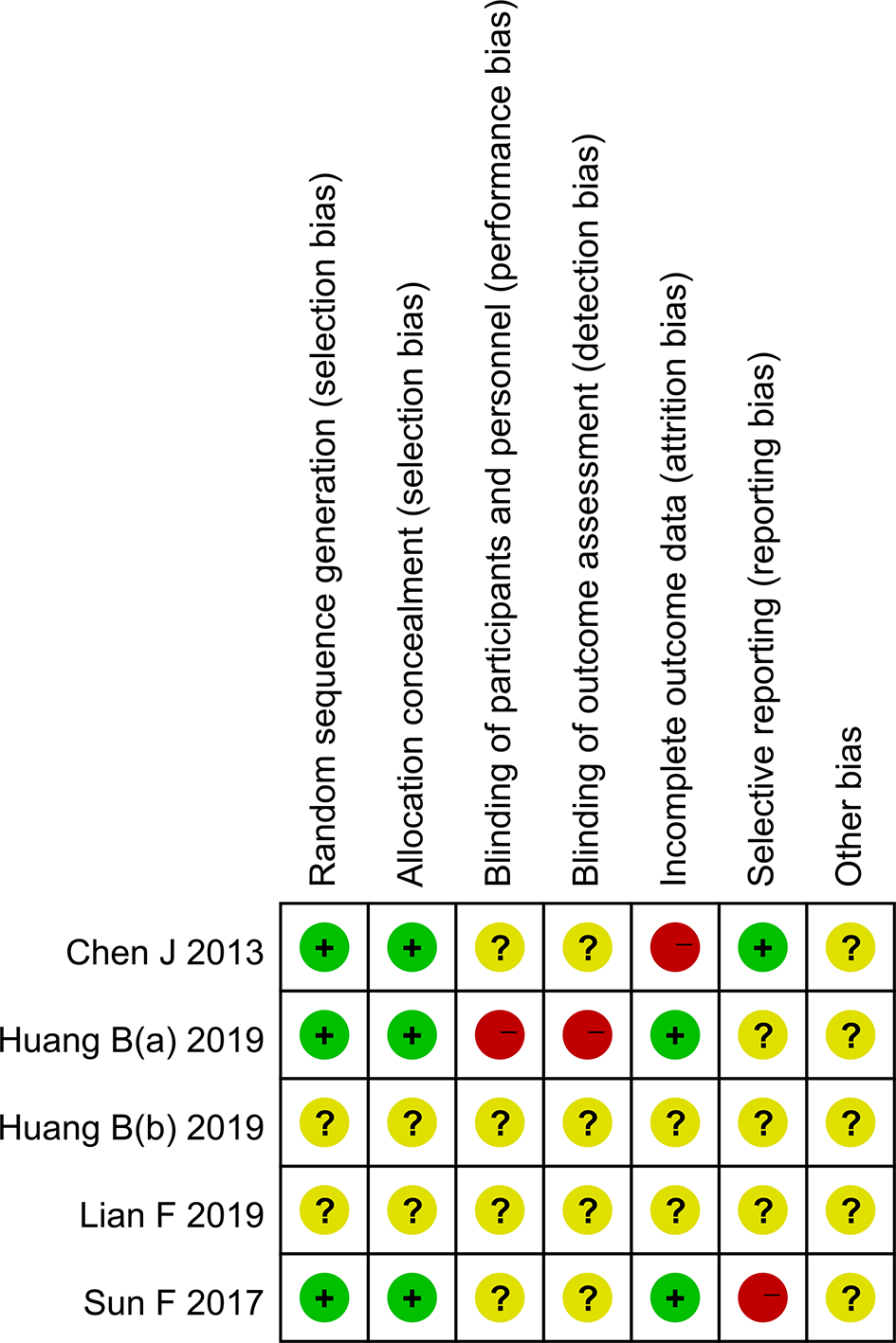
The risk of bias graph.

Three randomized clinical trials and two non-randomized clinical trials were included in the article. Among them, three randomized clinical trials described specific randomized protocols, but there was no clear expression for the blind method (**Figure 2**). Whether sample size was calculated was not described in the five studies. According to the NOS score, the scores of two non-randomized clinical trials were all above seven points, which belonged to low risk of bias. The outcomes of quality assessment were shown in **Table 3**. Publication bias was shown in the **Supplementary Material**.

**Table 3:** Risk of bias according to Newcastle-Ottawa Scale (NOS) in included retrospective studies.

Study	Representativeness of exposed cohort	Selection of non-exposed	Ascertainment of exposure	Outcome not present at start	Comparability	Assessment of outcome	Adequate follow-up	Adequacy of follow up	Overall
Lian F (18)	1	1	1	1	2	1	1	0	8
Huang B (b) (17)	1	1	0	1	2	1	1	0	7

Score of seven or more, four to six, and lower than four were considered to have low, moderate, and high risk of bias, respectively.

### Efficiency

#### OS

**Figure 3:**
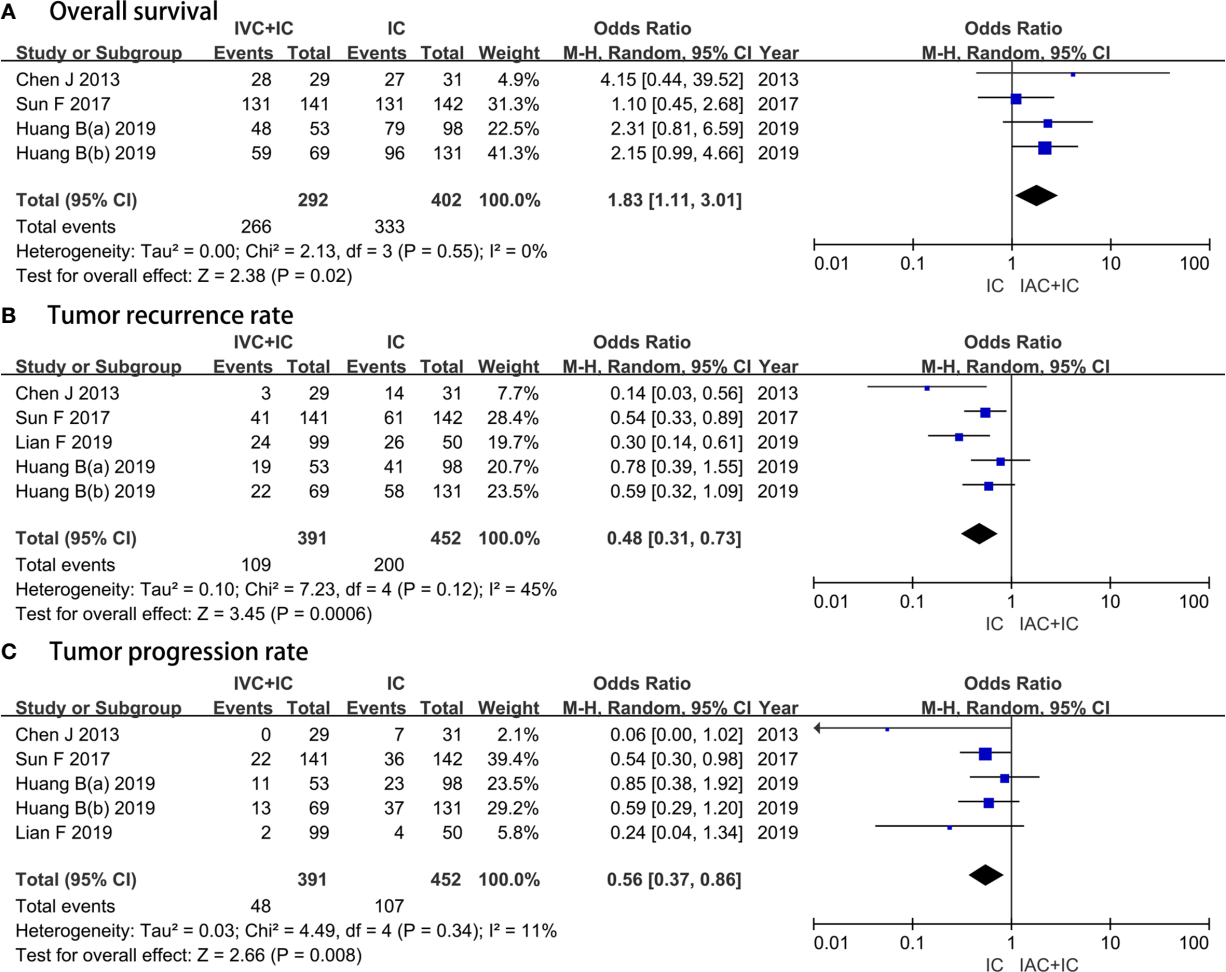
Forest plots showing the result of **(A)** overall survival, **(B)** tumor recurrence rate and **(C)** tumor progression rate. M–H, Mantel–Haenszel; CI, confidence interval; df, degrees of freedom.

Four studies with a sample of 694 patients evaluated the OS. The results of heterogeneity test were P = 0.55 and I^2^ = 0%. TRRs between the IAC + IC group and the IC group were 27.9% (109/391) and 44.2% (200/452), respectively. The forest plot indicated that the IAC + IC group had a statistically higher than the IC group in the OS (OR 1.83, 95%CI 1.11 to 3.01, P = 0.02) (**Figure 3A**).

#### TRR

Five studies with a sample of 843 patients evaluated the TRR. The results of heterogeneity test were P = 0.12 and I^2^ = 45%. TRRs between the IAC + IC group and the IC group were 27.9% (109/391) and 44.2% (200/452), respectively. The forest plot demonstrated that the IAC + IC group had a statistically lower than the IC group in the TRR (OR 0.48, 95%CI 0.31 to 0.73, P = 0.0006) (**Figure 3B**).

#### TPR

Five studies with a sample of 843 patients evaluated the TPR. The results of heterogeneity test were P = 0.34 and I^2^ = 11%. TPRs between the IAC + IC group and the IC group were 12.3% (48/391) and 23.6% (107/452), respectively. The forest plot identified that the IAC + IC group had a statistically lower than the IC group in the TPR (OR 0.56, 95%CI 0.37 to 0.86, P = 0.008) (**Figure 3C**).

#### RFS and PFS

**Figure 4:**
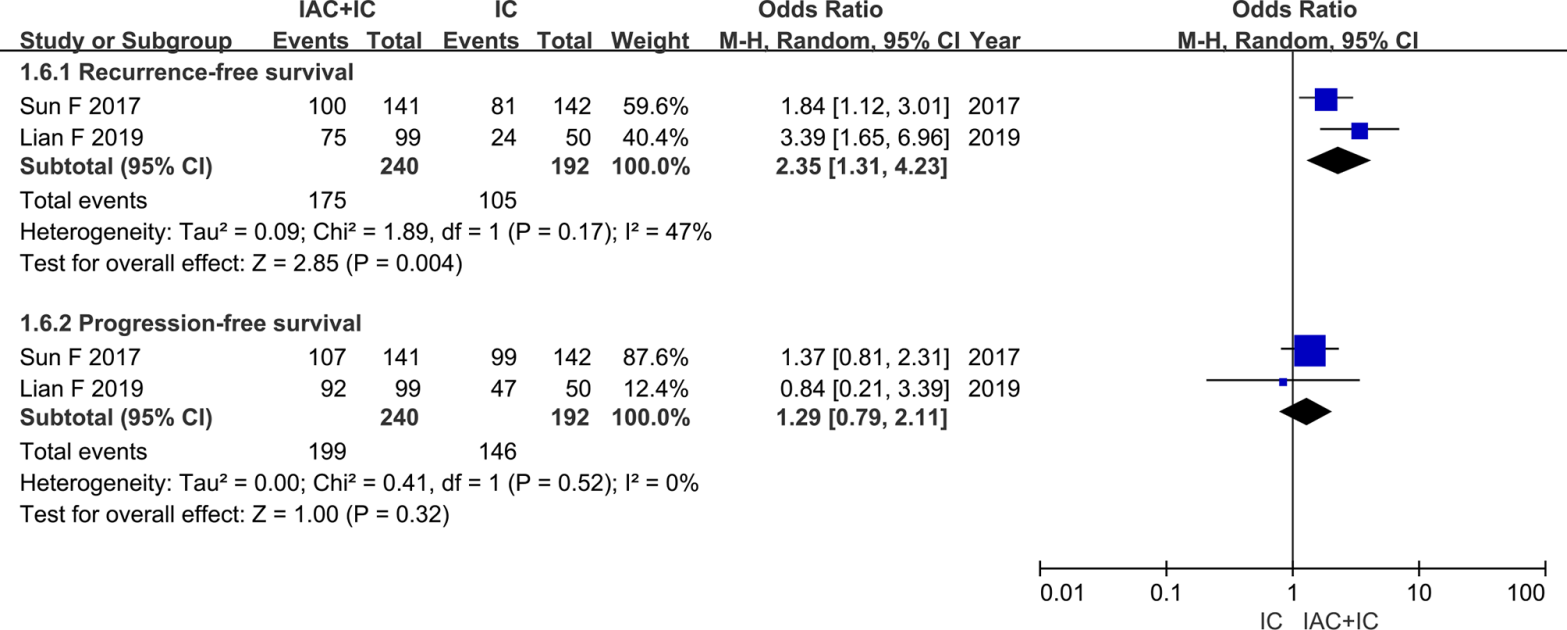
Forest plots showing the result of (1.6.1) recurrence-free rate and (1.6.2) progression-free survival. M–H, Mantel–Haenszel; CI, confidence interval; df, degrees of freedom.

Two studies included data on the RFS and PFS, gathering 432 patients. RFS between the IAC + IC group and the IC group was respectively 72.9% (175/240) and 54.7% (105/192). The results of heterogeneity test were P = 0.17 and I^2^ = 47%. PFS between the two groups was respectively 82.9% (199/240) and 76.0% (146/192). The results of heterogeneity test were P = 0.52 and I^2^ = 0%. The random-effects model indicated that the IAC + IC group had a statistically higher than the IC group in the RFS (OR 2.35, 95%CI 1.31 to 4.23, P = 0.004) and no statistical significance among the two groups in the PFS (OR 1.29, 95%CI 0.79 to 2.11, P = 0.32) (**Figure 4**).

#### TRI

**Figure 5:**
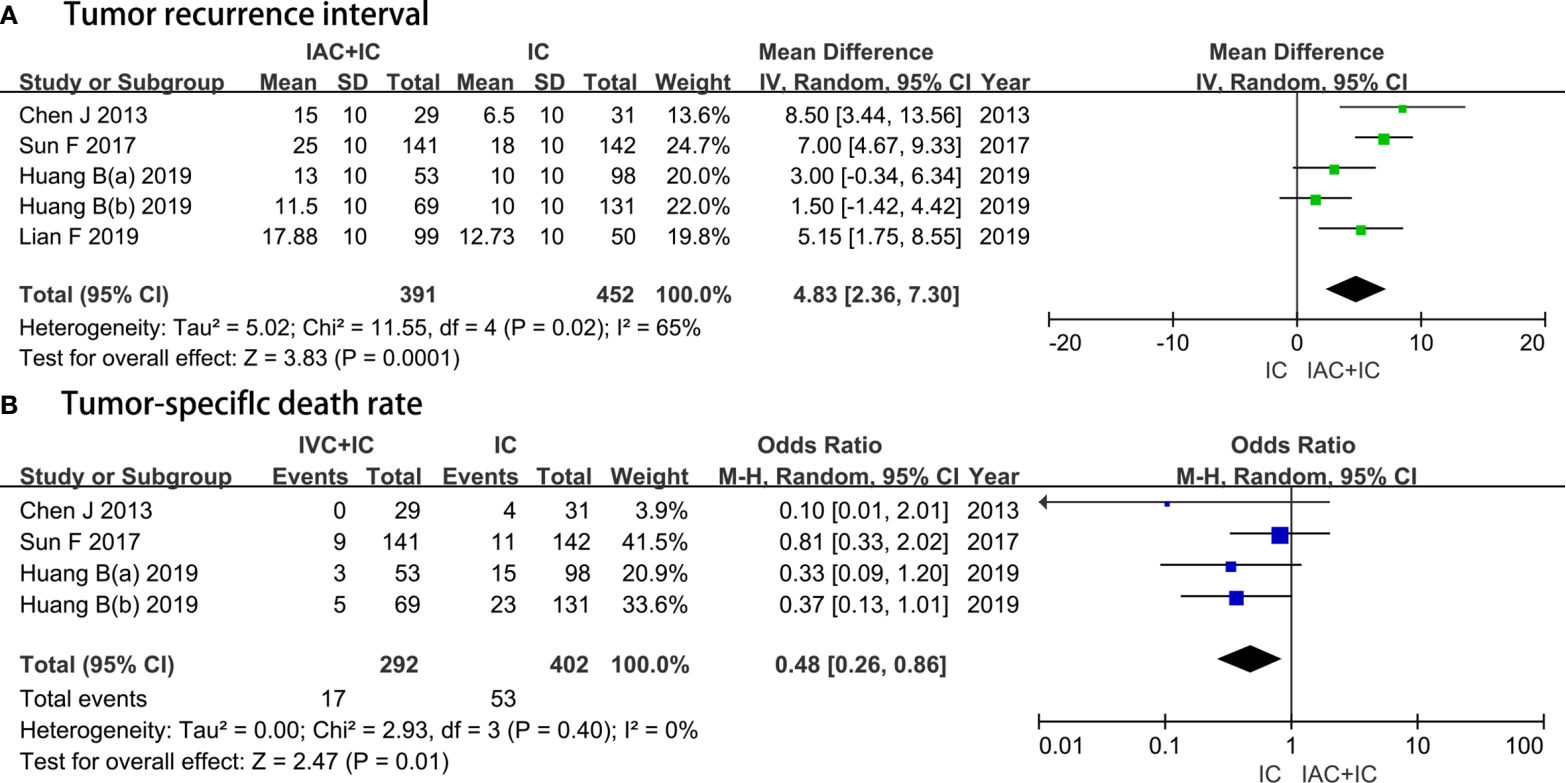
Forest plots showing the result of **(A)** tumor recurrence interval and **(B)** tumor-specific death rate. M–H, Mantel–Haenszel; CI, confidence interval; SD, standard deviation; IV, inverse variance; df, degrees of freedom.

Five studies with a sample of 843 patients evaluated the TRI. The results of heterogeneity test were P = 0.02 and I^2^ = 65%. The forest plots identified that the IAC + IC group had a greater effect compared with the IC group in extending the TRI (MD, 5.15; 95%CI, 1.75 to 8.55; P = 0.0001) (**Figure 5A**).

#### TSDR

Four studies with a sample of 694 patients evaluated the TSDR. The results of heterogeneity test were P = 0.40 and I^2^ = 0%. TSDR between the IAC + IC group and the IC group was 5.8% (17/292) and 13.2% (53/402), respectively. The forest plots identified that the IAC + IC group had a statistically lower than the IC group in the TSDR (OR 0.48, 95%CI 0.26 to 0.86, P = 0.01) (**Figure 5B**).

### Toxicity

Toxicities related with IAC were evaluated according to CTCAEv4.0 (**Table 4**). Generally, IAC was well tolerated by patients. Gastrointestinal toxicities were the most common AEs, including nausea/vomiting (49.0%). Hematological toxicities included hypoleukemia (11.3%), neutropenia (11.3%), alanine aminotransferase (19.2%), and creatinine (4.6%).

**Table 4:** Intra-arterial chemotherapy-related side effects.

Side effects	Study	Grade 0 No	Grade INo	Grade II No	Grade III No	Grade IV No	Incidence%
Nausea/vomiting	Huang B (a) (16)	18	18	9	8	–	66.0
	Huang B (b) (17)	29	21	10	9	–	57.9
	Sun F (10)	–	36	17	0	0	37.6
	Chen J (9)	14	11	3	1	0	51.7
Hypoleukemia	Huang B (a) (16)	46	4	1	2	–	13.2
	Huang B (b) (17)	62	4	1	2	–	11.3
	Chen J (9)	26	2	1	0	0	10.3
Neutropenia	Huang B (a) (16)	44	6	1	2	–	16.9
	Huang B (b) (17)	62	3	3	–	1	11.3
	Sun F (10)	–	11	3	0	0	9.9
	Chen J (9)	26	2	0	0	1	10.3
Alanine aminotransferase	Huang B (a) (16)	41	10	1	1	–	22.6
	Huang B (b) (17)	57	8	3	1	–	17.4
	Chen J (9)	24	4	1	0	0	17.2
Creatinine	Huang B (a) (16)	66	1	2	–	–	4.5
	Huang B (b) (17)	67	1	1	–	–	2.3
	Chen J (9)	27	2	0	0	0	6.9

**Figure 6:**
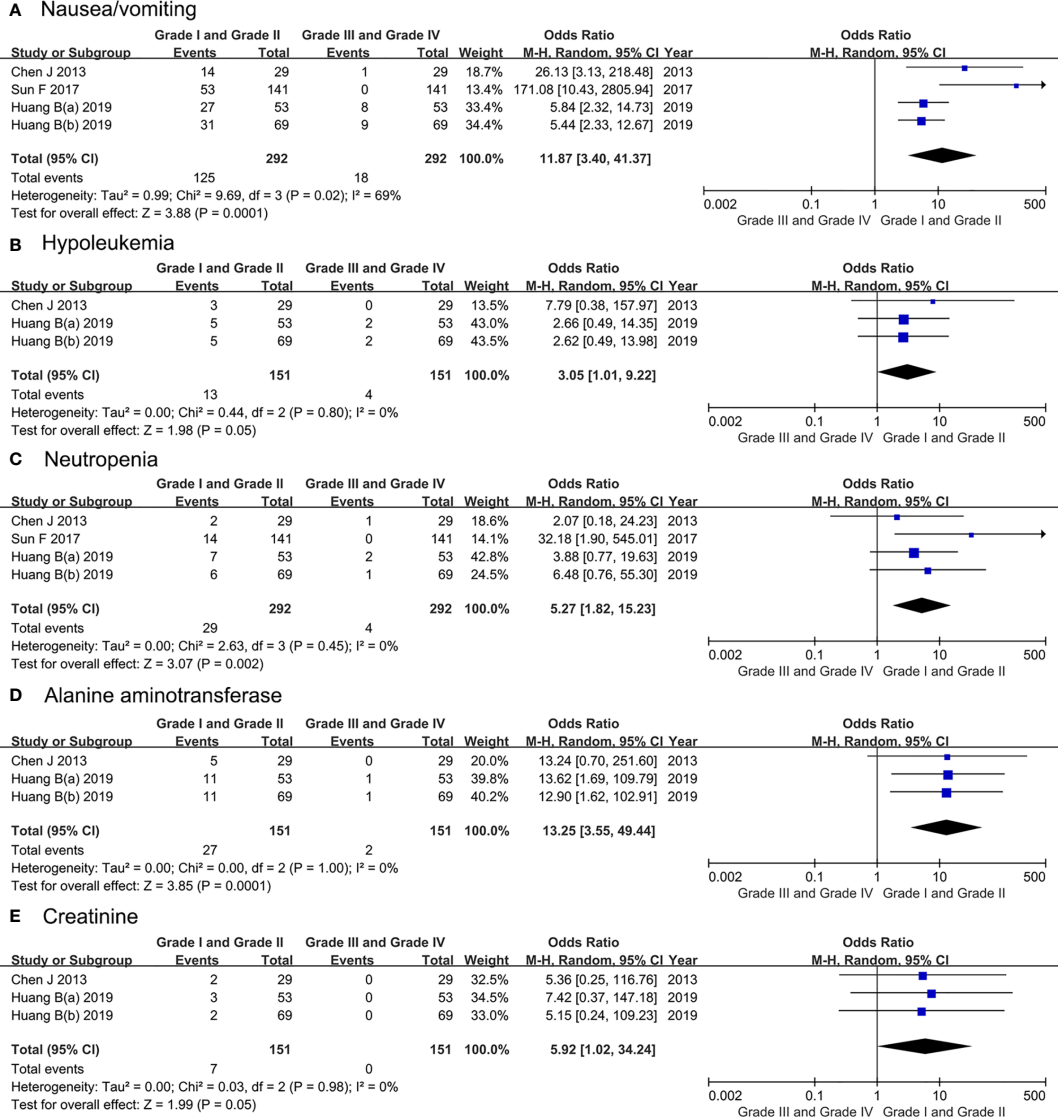
Forest plots showing the result in IAC related adverse events including **(A)** nausea/vomiting, **(B)** hypoleukemia, **(C)** neutropenia, **(D)** alanine aminotransferase, and **(E)** creatinine. M–H, Mantel–Haenszel; CI, confidence interval; df, degrees of freedom.

Most toxicities were mild and reversible (grades 1–2, 22.3% *vs*. grade 3–4, 2.7%). Most toxicities were grades 1–2 rather than grades 3–4. **Figure 6** showed whether there was a remarkable difference between grades 1**-**2 and grades 3–4 about every AE. The forest plots identified that grades 1–2 had a higher incidence than grades 3–4 in terms of nausea/vomiting (OR 11.87, 95%CI 3.40 to 41.37, P = 0.0001), neutropenia (OR 5.27, 95%CI 1.82 to 15.23, P = 0.002) and alanine aminotransferase (OR 13.25, 95%CI 3.55 to 49.44, P = 0.0001). However, there was no significant difference between grades 1–2 and grades 3–4 in number of hypoleukemia (OR 3.05, 95%CI 1.01 to 9.22, P = 0.05) and creatinine (OR 5.92, 95%CI 1.02 to 34.24, P = 0.05).

## Discussion

According to the risk of tumor recurrence and progression, European Association of Urology (EAU) divided superficial BC into three groups (19). Multiple T1G2 tumor, Ta-T1G3 tumor with or without Cis, and Cis alone are classified as HRBC; multifocal T1G1, TaG2, and single T1G2 tumor are classified as medium-risk BC; TaG1, single T1G1 tumor are classified as low-risk BC (20, 21). Due to the high recurrence rate and the high risk of progression to muscle invasive bladder cancer (MIBC), previous studies recommended early RC for the treatment of superficial HRBC (22, 23). However, some literatures have shown that early RC was an over-treatment with the more AEs for patients including urinary incontinence, erectile dysfunction and some psychological problems. And studies have reported that there was no significant difference in OS between early RC and initial TUR plus adjuvant IC (24, 25). The study by Li et al. reported that intravenous chemotherapy was as effective as RC for MIBC (26).

With the regeneration of drugs, IAC has become one of the most important options for HRBC, and many studies have reported some promising results (27–30). Moreover, clinical phase III trial reported that the IAC of gemcitabine and cisplatin (GC) regimen had similar survival advantages as methotrexate, vinblastine, adriamycin, and cisplatin (MVAC) regimen, but it had a better tolerance in locally advanced and metastatic BC (31). So, IAC with GC regimen has become the first choice for patients with locally advanced or metastatic BC. Recently, some articles have reported that bladder preservation rate of cisplatin-based IAC was more than 80% for patients with locally advanced BC (32, 33). Based on these results, we believed that IAC could reduce the recurrence and progression of tumor and significantly improve the bladder preservation rate for patients with superficial HRBC after first TUR or partial cystectomy.

This meta-analysis aimed to access the efficacy of IAC + IC *versus* IC alone for patients with HRBC after bladder-sparing surgery. The results found that the IAC + IC group had a greater improvement of OS and significant reduction in terms of TRR and TPR compared with the IC group. The RFS in the IAC + IC group was significantly higher than that in the IC group, but there was no significant difference in PFS between the two groups. Moreover, the combination of IAC and IC significantly extended the TRI and reduced the TSDR for patients with HRBC compared with IC alone.

As a novel strategy, the efficacy and safety of IAC are worthy of attention. Although IAC/IC has been proved to be promising in preventing tumor recurrence in our study, IAC/IC did not significantly reduce tumor progression compared with IC alone, which was inconsistent with the result reported by Sun et al. (10) and Chen et al. (9). However, one RCT reported that almost all the patients with progression were multifocal high-risk tumors, and the only moderate-risk patients with progression were also multifocal tumors (18). Moreover, the progression rate in the IAC group was extremely low, and only two patients presented progression, suggesting that IAC may have a preventive effect on the recurrence and progression of multifocal tumors (18).

The theory of IAC in treating HRBC is mainly derived from its effectiveness in neoadjuvant chemotherapy and adjuvant chemotherapy of MIBC (34, 35). A previous study has proved that intravenous chemotherapy by GC achieved some results in bladder reservation therapy for T1G3 BC patients (36). By the multivariate analysis model, Liu et al. (37) identified some prognostic factors and compared cancer specific survival rate relative to early RC, concluding that 79.0% of patients in the GC group had successful bladder preservation.

The idea of IAC is to inject chemotherapeutic drugs directly into the blood vessels leading to the neoplastic organs, so that the concentration of antitumor drugs in the neoplastic organs will be higher, achieving the more effective distribution (38, 39). However, adverse reactions related with IAC should not be ignored. In our study, although about half of the patients experienced some toxicities, most of them were mild (grades 1–2, 22.3% *vs.* grades 3–4, 2.7%) and easily reversible without extensive hospital intervention. The forest plots identified that grades 1–2 had a higher incidence than grades 3–4 in terms of nausea/vomiting, neutropenia and alanine aminotransferase. However, there was no significant difference between grades 1–2 and grades 3–4 in number of hypoleukemia and creatinine.

Other complications related with IAC included infection, hematoma, thrombosis, *etc.*, but complications related with puncture were not reported in the included studies. Most of the side effects were minor and could be easily managed by symptomatic treatment. Many studies also reported IAC as a safe therapy with limited and minor side effects (8, 9, 40). Is IAC over-treatment for patients with superficial HRBC because of its AEs? Some literatures have reported the safety and toxicity of systemic chemotherapy and found that AEs in the form of myelosuppression, renal dysfunction, and metabolic disorders were temporary and moderate. In two of the studies, the major grades 3–4 hematological toxicity was neutropenia and the major grades 3–4 non-hematological toxicity was anorexia (31, 41).

Currently, bladder preservation is becoming a hot topic. Immune checkpoint inhibitors have increasingly become a therapeutic option for many solid tumors (42, 43). In BC, high expression of anti-programmed cell-death protein 1 (PD-1) and programmed death-ligand 1 (PD-L1) have been found to be closely related with advanced and aggressive tumors with lower survival rate (44, 45). In this case, PD-1/PD-L1 inhibitors combined with or without BCG are being validated in NMIBC with promising results. The rationale was that PD-L1 expression has been associated with increased resistance to BCG immunotherapy. Moreover, granulomas induced by BCG in BCG-unresponsive patients showed high levels of PD-L1 expression. The high expression of PD-L1 may suppress the T-cell response induced by BCG and be the cause of the BCG failure (46, 47). However, a meta-analysis performed by Bersanelli et al. showed that the current evidence does not support a statistically significant effect from immune checkpoint inhibitors over the standard treatment for advanced upper tract urothelial carcinoma (48). Szabados et al. identified that immune checkpoint inhibitor monotherapy is not superior to chemotherapy as things currently stand, and the chemo-immunotherapy combination showed a probable efficacy signal, but this appeared to be insufficient to change practice (49). Huang et al. reported that IAC combined with IC used in high-risk NMIBC could reduce the recurrence and progression as effective as BCG instillation with lower adverse events (50).

In summary, the combination of IAC and IC can be an option for patients with HRBC. With the further study of molecular mechanism of BC, the treatment mode of HRBC still needs further study. We need to acknowledge the limitations of this analysis. The quality of the included studies is insufficient, mainly in the aspects of study design, patient selection and result data extraction. In addition, the bias of selection factors and subjective factors may also affect the final results of this study. Therefore, the results of this study should be interpreted with caution. The advantages and disadvantages between IAC + IC and IC alone still need to be verified by RCTs with larger sample sizes.

## Conclusions

Patients with HRBC treated with IAC + IC after bladder-sparing surgery had a significant improvement in overall survival, recurrence-free survival, time interval to first recurrence, tumor recurrence rate, tumor progression rate and tumor-specific death rate than patients treated with IC alone. However, progression-free survival was not significantly correlated with treatment strategy. In addition, patients seemed to tolerate well the toxicities related with IAC.

## Data Availability Statement

The original contributions presented in the study are included in the article/**Supplementary Material**. Further inquiries can be directed to the corresponding authors.

## Author Contributions

Conceptualization: YZ. Data curation: ZZ, YC, and SH. Formal analysis: ZZ, YC, SH, and ZC. Funding acquisition: YZ. Investigation: ZZ and YC. Methodology: ZZ, YC, and ZC. Project administration: YZ. Resources: ZZ and YC. Software: ZZ, YC, and SH. Supervision: YZ and ZC. Writing—original draft: ZZ and YC. All authors contributed to the article and approved the submitted version.

## Funding

This work was supported by Beijing Municipal Administration of Hospitals’ Ascent Plan, Code: DFL20190502, Beijing Municipal Administration of Hospitals Clinical Medicine Development of Special Funding Support, Code: ZYLX201820, and National Nature Science Foundation of China, Code: 81801429.

## Supplementary Material

The Supplementary Material for this article can be found online at: https://www.frontiersin.org/articles/10.3389/fonc.2021.651657/full#supplementary-material

## Conflict of Interest

The authors declare that the research was conducted in the absence of any commercial or financial relationships that could be construed as a potential conflict of interest.
