# Supplementary material for: The Efficacy of Intra-Arterial Plus Intravesical Chemotherapy Versus Intravesical Chemotherapy Alone After Bladder-Sparing Surgery in High-Risk Bladder Cancer: A Systematic Review and Meta-Analysis of Comparative Study
Source: Front Oncol. 2021 May 27;11:651657. doi: 10.3389/fonc.2021.651657 (PMC8190377; doi:10.3389/fonc.2021.651657)
Supplement: Supplementary file 1 [file DataSheet_1.pdf]

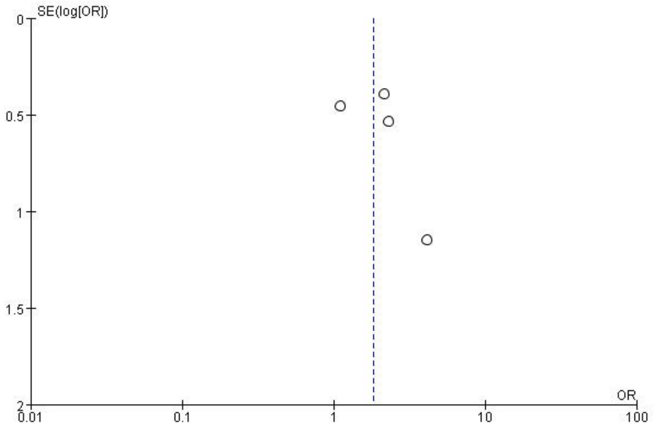

1. overall survival

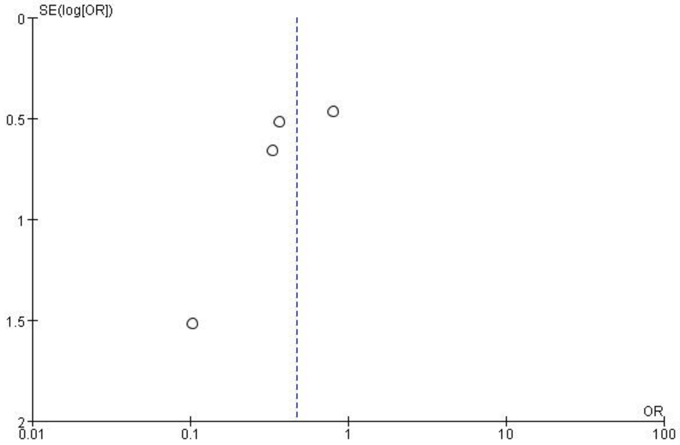

2. tumor-specific death rate

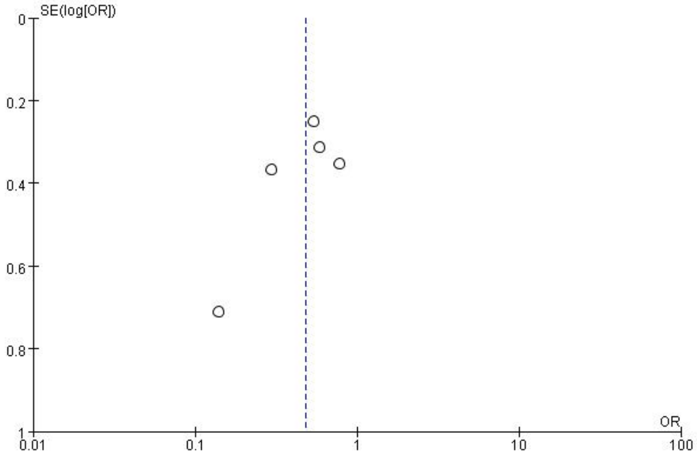

3. tumor recurrence rate

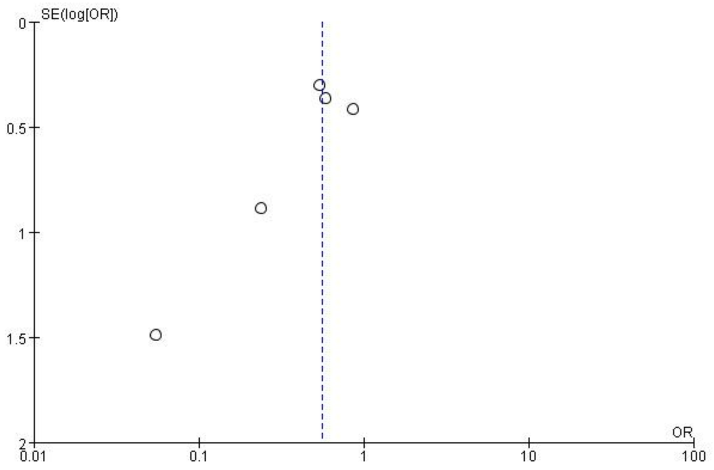

4. tumor progression rate

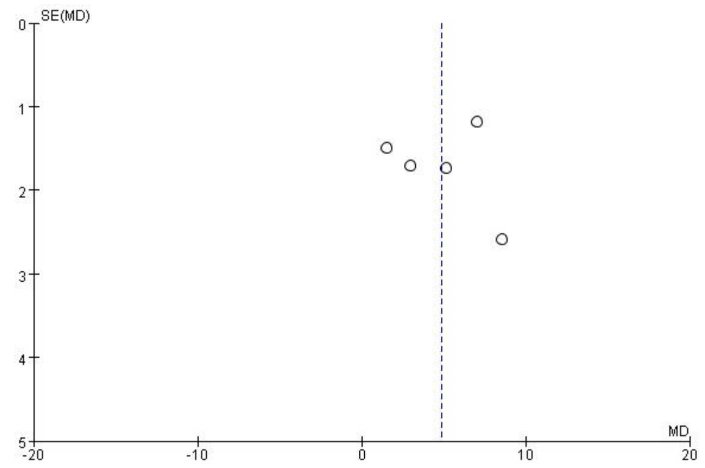

5. tumor recurrence interval

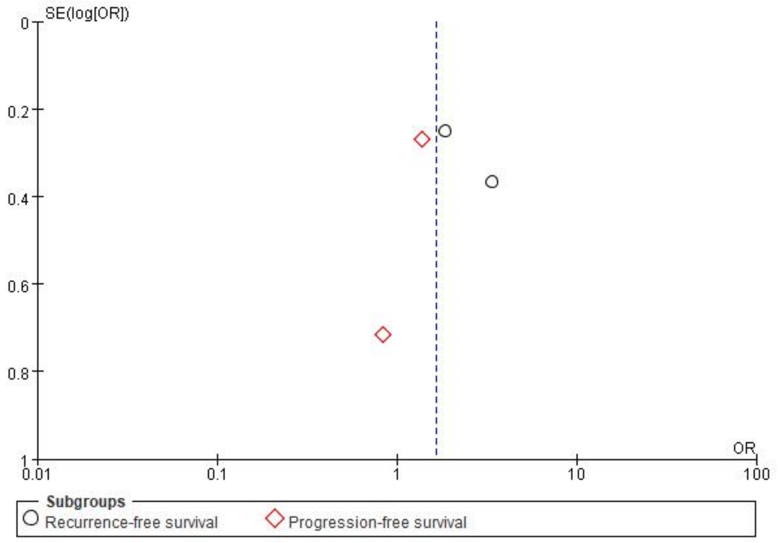

6. recurrence-free survival and progression-free survival

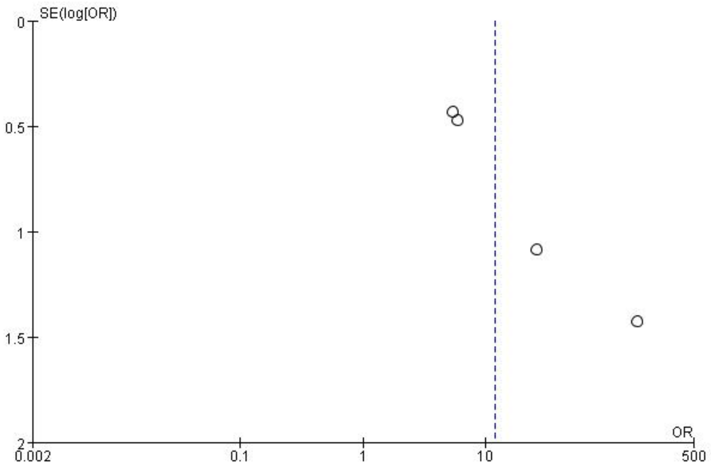

7. nausea/vomiting

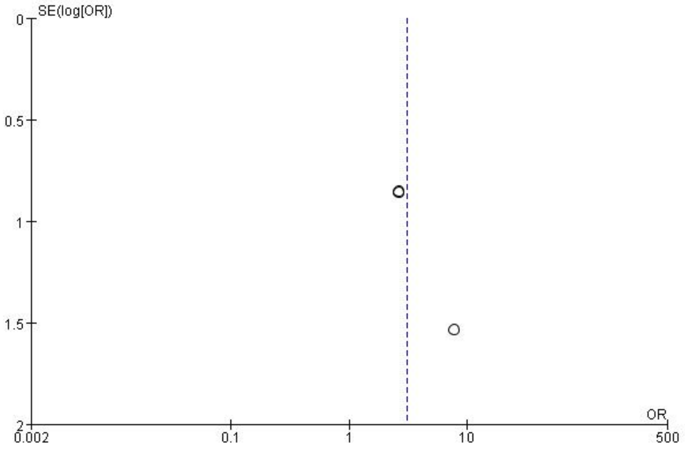

8. hypoleukemia

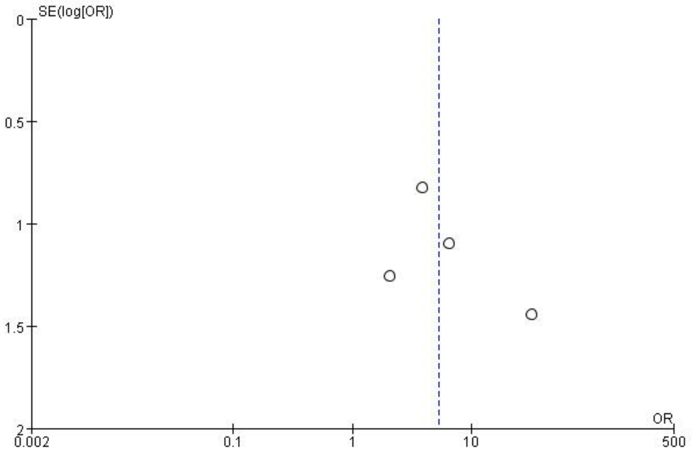

9. neutropenia

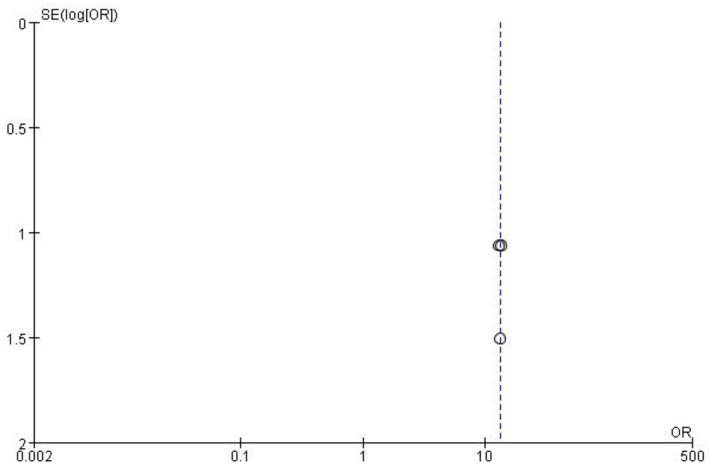

10. alanine aminotransferase

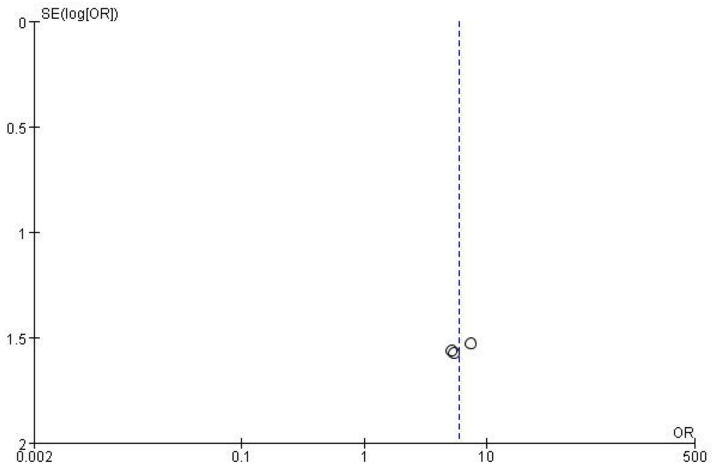

11. creatinine
